# Winery By-Products as Sources of Bioactive Tryptophan, Serotonin, and Melatonin: Contributions to the Antioxidant Power

**DOI:** 10.3390/foods12081571

**Published:** 2023-04-07

**Authors:** Nieves Baenas, Cristina García-Viguera, Raúl Domínguez-Perles, Sonia Medina

**Affiliations:** 1 Department of Food Technology, Food Science and Nutrition, Faculty of Veterinary Sciences, Regional Campus of International Excellence “Campus Mare-Nostrum”, Campus de Espinardo, University of Murcia, 30100 Murcia, Spain; 2 Laboratorio de Fitoquímica y Alimentos Saludables (LabFAS), Departamento de Ciencia y Tecnología de, Alimentos, CEBAS-CSIC, Campus of the University of Murcia-25, Espinardo, 30100 Murcia, Spain

**Keywords:** grape stems, grape pomace, wine lees, FRAP, ABTS, ORAC

## Abstract

The amino acid tryptophan and its derived molecules serotonin and melatonin are involved in a wide range of physiological functions that contribute significantly to human health, namely antioxidant, immune-active, and neurological properties. Grapes and wine are a source of these compounds, but their presence in wine by-products remains underexplored. Therefore, the aim of this work was the identification and quantification of tryptophan, serotonin, and melatonin in winery by-products (grape stems, grape pomace, and wine lees) by ultra-high performance liquid chromatography coupled to electrospray ionization and mass spectrometer with triple-quadrupole technology (UHPLC-ESI-QqQ-MS/MS), as well as the evaluation of the extracts obtained (by applying specific extraction conditions for each of them) for their antioxidant and reducing capacity (by three different and complementary methods: FRAP, ABTS^•+^, and ORAC). Furthermore, correlation analyses were developed to establish the contribution of the different analytes to the total antioxidant activity. The main results obtained pointed out grape stems as the by-product with the highest tryptophan content (96.28 mg/kg dw) and antioxidant capacity (142.86, 166.72, and 363.24 mmol TE/kg dw, FRAP, ABTS^•+^, and ORAC, respectively), while serotonin and melatonin were the predominant derivatives in grape pomace (0.086 and 0.902 µg/kg dw, respectively). The antioxidant capacity of the standards was also analysed at the concentrations found in the matrices studied. A significant correlation was found between the concentration of the pure tryptophan standard and the antioxidant capacity (ABTS^•+^, r^2^ = 0.891 at *p* < 0.001 (***); FRAP, r^2^ = 0.885 at *p* < 0.01 (**); and ORAC, r^2^ = 0.854 at *p* < 0.01 (**)). According to these results, winery by-products can be highlighted as valuable materials to be used as novel ingredients containing tryptophan, serotonin, and melatonin, while tryptophan was identified as the most relevant contributor (out of phenolic compounds) to the antioxidant capacity exhibited by wine by-products.

## 1. Introduction

**Figure 1:**
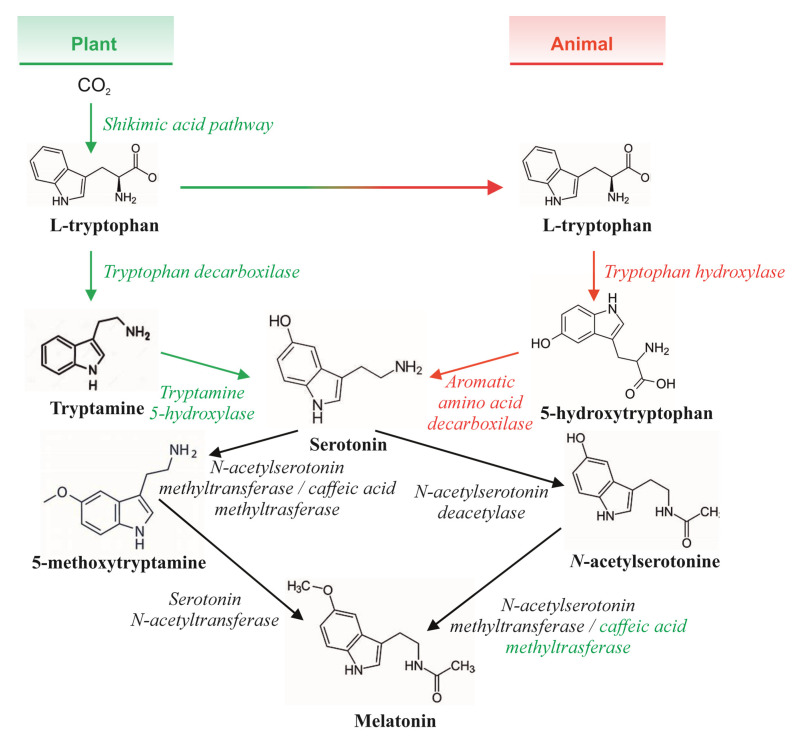
In humans, the essential amino acid tryptophan is used to synthesise serotonin and melatonin.

The human hormone *N*-acetyl-5-methoxytryptamine (commonly known as melatonin) is an indoleamine produced by the pineal gland via the synthetic pathway from the essential amino acid tryptophan. This amino acid is enzymatically converted to 5-hydroxytryptamine, also known as serotonin, by tryptophan-5-hydroxylase and 5-hydroxytryptophan decarboxylase, and then melatonin is enzymatically synthesised from serotonin with the concourse of serotonin *N*-acetyltransferase and hydroxy indole-*O*-methyltransferase [1] (Figure 1). Once synthesised, serotonin and melatonin are secreted by the pineal gland and distributed throughout the organism to exert specific functions. With regard to melatonin, its bioactivities are linked to a number of biological processes, including the regulation of circadian rhythms and the regulation of various neuroendocrine, cardiovascular, and immune functions [2]. Serotonin is also responsible for critical functions in human physiology, closely linked to proper cardiovascular, respiratory, thermoregulatory, and immune health, and is associated with feeding behaviour, regulation of the circadian rhythms (sleep-wake cycle), or pain sensitivity, and is at the basis of several neurological disorders (e.g., Parkinson’s and Alzheimer’s diseases) [3]. In addition to these physiological functions, serotonin and melatonin have been described as being able to protect cellular components, such as DNA, membrane lipids, and/or cytosolic proteins, from the deleterious effects caused by free radicals and reactive oxygen species (ROS), which have been related to the onset and progression of different chronic pathophysiological conditions [4], attracting the attention of the scientific community in recent years.

Alterations on the physiological levels of tryptophan derivatives have been related to a number of pathological conditions. In this context, the usefulness of administering herbal medicines has been identified in the last few decades as an alternative to correcting deficiencies of essential compounds and as an affordable treatment to prevent functional disorders [5]. In this context, the identification of exogenous sources of essential molecules for the proper development of physiological responses (e.g., tryptophan, serotonin, or melatonin) has become a necessity. Thus, phytomelatonin is the term used to describe melatonin of plant origin. Since its first description in higher plants, over two decades ago, several studies have reported its presence in a variety of plant tissues (leaves, fruits, stems, roots, flowers, sprouts, and seeds), including both edible and ornamental (non-edible) plant species [6,7]. The modulatory effect of the plant physiology of melatonin has led to the investigation of its ability to increase the yield and quality of fruit and vegetable production concerning pomegranate, apricot, mango, and sweet cherry, among others, as well as their shelf life and behaviour during storage [8,9,10,11].

In 2011, phytomelatonin was detected for the first time as a new bioactive compound in wine from several grape varieties and various grape-related foods, such as grape juice, grape must, and grappa (Italian grape pomace brandy) [12]. According to Jiang-Fei et al., melatonin could be synthesised in these matrices by yeast during alcoholic fermentation and by lactic acid bacteria (LAB) during malolactic fermentation, or from grape berries via a common synthetic pathway from tryptophan [13]. This is of special relevance because, in addition to wine, melatonin could also be detected in several by-products derived from the presence of tryptophan and serotonin. In this sense, the wine industry is associated with economic and environmental handicaps due to the production of huge amounts of solid (grape stems and grape pomace) and semi-solid (wine lees) pollutant residues, which represent up to 30% of the total material processed. This point is a worrying fact for the main wine-producing countries such as Spain, France, and Italy, among others [14]. In this sense, the identification of the role of winery by-products as a source of bioactive tryptophan derivatives would allow for new valorisation opportunities, which are of particular interest because they constitute inexpensive sources of bioactive phytochemicals, including serotonin and melatonin, which have not yet been explored. Thus, increasing the current knowledge of the phytochemical profile of such materials will boost the sustainability of the wine sector, with consequent economic, social, and environmental benefits.

Based on these antecedents, this study aims to uncover the value of grape stems, grape pomace, and wine lees as dietary sources of melatonin and its precursor molecules tryptophan and serotonin, as well as the relative contribution of these compounds to the total antioxidant power of the aforementioned winery by-products. With this objective, the tryptophan, serotonin, and melatonin dedicated extracts were evaluated for their content of bioactive molecules and their antioxidant capacity by three complementary methods (ferric reducing antioxidant power (FRAP), 2,2′-azino-bis (3-ethylbenzothiazoline-6-sulphonic acid radical (ABTS^•+^) scavenging, and oxygen radical absorbance capacity (ORAC)). The correlation analysis between the tryptophan, serotonin, and melatonin contents in the winery by-products and the antioxidant capacity results was performed to provide consistent evidence of the functionality of these compounds. To further demonstrate the actual contribution of these molecules to the antioxidant power of the winery by-products, authentic standards of the target bioactive compounds, at matching concentrations relative to the winery by-products’ extracts, were analysed for antioxidant behaviour.

## 2. Materials and Methods

### 2.1. Chemicals and Reagents

Standards of tryptophan, serotonin, and melatonin, as well as the Bis-Tris reagent, ABTS^•+^, 2,4,6-tripyridyl-s-triazine (TPTZ), fluorescein, 2,2′-azobis(2-methylpropionamidine) (AAPH), ferric (III) chloride hexahydrate, manganese (IV) oxide, and the standard 6-hydroxy-2,5,7,8-tetramethylchroman-2-carboxylic acid (Trolox) were obtained from Sigma-Aldrich (Steinheim, Germany). The 6-aminoquinolyl-N-hydroxysuccinimidyl carbamate (AQC) reagent was purchased from Chemos GmbH (Regenstauf, Germany). Formic acid, hydrochloric acid (37%), dipotassium hydrogen phosphate, sodium acetate, and ammonium acetate were purchased from Panreac Quimica S.A. (Castellar del Vallés, Barcelona, Spain). Boric acid was purchased from Probus (Badalona, Barcelona, Spain). All LC-MS-grade solvents were obtained from JT Baker (Phillipsburg, NJ, USA). Ultrapure water was produced using a Millipore water purification system (Darmstadt, Germany).

### 2.2. Plant Material and Extracts

Winery by-products: stems and pomace, and wine lees, were obtained from the winery industry Bodegas Viña Elena S.L. (Jumilla, Murcia, Spain), after wine production (*Vitis vinifera* L. var. ‘Monastrell’). For analytical purposes, the sampled materials corresponding to three batches (grape stems, grape pomace, and wine lees) from the 2021 season were thoroughly mixed and bulked into four replicates per residue (*n* = 4). The by-product samples were freeze-dried up to a constant weight using a CHRIST 2-4D vacuum concentrator (Wolflabs, York, UK). The dry materials were ground into a fine powder and stored in the dark for further phytochemical and radical scavenging analyses.

### 2.3. Derivatization and Analysis of Tryptophan by UHPLC-ESI-QqQ-MS/MS

The tryptophan content was quantified in the winery by-products (grape stems, grape pomace, and wine lees) using the method previously described by Collado-González et al. (2014) [15]. Briefly, to obtain the amino acid extract, samples (20 mg of powder) were homogenised with 500 µL of MeOH/deionised water (50:50, *v*/*v*). Afterwards, these solutions were vortexed for 30 s, incubated for 5 min on ice, and sonicated for 10 min at 20 °C in an ultrasound bath. The homogenates were centrifuged (with centrifuge 5804 R, Hamburg, Germany) for 10 min at 17,900× *g* and 4 °C. The collected supernatant was derivatised immediately using AQC as the derivatisation reagent, according to Salazar et al. (2012) [16]. To complete the derivatisation reaction, 350 μL of borate buffer (0.2 M sodium borate, pH 8.8) was mixed with 50 μL of the amino acid’s standard or the winery by-products’ extract and 100 μL of 10 mM AQC in acetonitrile. The vial was vortexed and allowed to stand for 1 min at room temperature. Subsequently, the vial was heated to 55 °C for 10 min.

The identification and quantification of tryptophan were performed by UHPLC-ESI-QqQ-MS/MS on an AccQ Tag Ultra BEH column (2.1 × 100 mm, 1.7 μm) (Waters Corp., Dublin, Ireland) using a UHPLC system coupled with a 6460 tandem mass spectrometer (Agilent Technologies, Waldbronn, Germany) operated according to the chromatographic and ionisation specifications described by Collado-González et al. (2014) [15]. The mobile phases used were 50 mL of an aqueous solution (acetonitrile/formic acid/5 mM acetate ammonium (10:6:84, *v*/*v*/*v*) diluted with 950 mL deionised water (Solvent A) and acetonitrile/formic acid (99.9:0.1, *v*/*v*) (solvent B), and the flow rate was 0.5 mL/min and the injection volume was 20 µL. Data acquisition and processing were carried out by the MassHunter software version B.08.00 (Agilent Technologies, Waldbronn, Germany).

### 2.4. Extraction of Serotonin and Melatonin and Analysis by UHPLC-ESI-QqQ-MS/MS

Each sample (100 mg) was mixed with 750 µL of methanol. The samples were then vortexed for 1 min and stored overnight at 4 °C. Afterwards, the samples were centrifuged at 11,900× *g* for 5 min at 4 °C (model EBA 21, Hettich Zentrifugen, Sigma-Aldrich (Steinheim, Germany)). The supernatant was collected and dried using a SpeedVac concentrator (Savant SPD121P, ThermoScientific, Madrid, Spain). The dry extracts were reconstituted with 150 µL of methanol/deionised water (80:20, *v*/*v*), filtered through a 0.22-μm PVDF filter (13 mm) (Millex HV13, Millipore, Bedford, MA, USA), and stored at 4 °C until analysis.

The chromatographic separation of serotonin and melatonin was performed using a UHPLC coupled with a 6460 triple quadropole-MS/MS (Agilent Technologies, Waldbronn, Germany) and a BEH C18 analytical column (2.1 × 50 mm, 1.7 µm). Chromatographic conditions were set using deionised water/formic acid (99.9:0.1, *v*/*v*) (solvent A) and acetonitrile/formic acid (99.9:0.1, *v*/*v*) (solvent B). The chromatographic separation of the target compounds was performed according to the following gradient (time (minutes), %B): (0, 40%); (1.50, 40%); (1.51, 90%); (3.50, 90%); (3.51, 40%); (4.50, 40%). The injection volume and flow rate were 18 µL and 0.3 mL/min, respectively. Data were acquired using the MassHunter software version B.08.00 (Agilent Technologies, Waldbronn, Germany).

The identification and quantification of tryptophan, serotonin, and melatonin were performed by resorting to the specific transitions and calibration curves of authentic standards freshly prepared each day of analysis (Table 1).

### 2.5. Determination of the Antioxidant Activity by In Vitro Tests

The antioxidant capacity of the analytical extracts prepared specifically for the determination of tryptophan, as well as serotonin and melatonin, in grape stems, grape pomace, and wine lees was quantified by three different and complementary methods adapted to a microscale: FRAP [17], ABTS^•+^ [18], and ORAC [19]. Briefly, for FRAP analysis, 20 µL of the sample/standard/blank and 180 µL of the FRAP reagent were added to each well, and the reaction was read at 593 nm after 4 min of incubation. The FRAP reagent was freshly prepared by mixing a 10 mM TPTZ solution in 40 mM HCl (37%), 20 mM FeCl_3_·6H_2_O solution, and 0.3 M acetate buffer (pH 3.6) in the ratio of 1:1:10 (*v*/*v*/*v*). For ABTS^•+^ analysis, the reaction was started by adding 50 μL of the diluted sample/standard/blank to each well containing 200 μL of the free radical (ABTS^•+^)-activated solution. The samples were read at 734 nm after 20 min of incubation. For the ORAC method, 20 µL of the sample/standard/blank and 200 µL of fluorescein (5 µM) were added to each well. Then, the AAPH solution (20 µL) was added to start the reaction. Fluorescence was recorded every 5 min for 120 min using an excitation wavelength of 485 nm and an emission wavelength of 528 nm. ORAC values were calculated as the difference in areas under the fluorescein decay curve (AUC) between the blank and a sample. Assays were measured by using 96-well microplates in a microplate spectrophotometer (BioTek Instruments, Winooski, VT, USA). Trolox was used as the standard, and the results were expressed as mmol of Trolox equivalents (TE)/kg sample dry weight (dw) or µmol TE/kg of dw for tryptophan-extracted samples or serotonin- and melatonin-extracted samples, respectively.

### 2.6. Statistical Analysis

The experimental design included four randomised replicates (*n* = 4) for each plant material (grape stems, grape pomace, and wine lees), and the results were presented as mean ± standard deviation (SD). All data exhibited normal distribution and homogeneity of variance (Shapiro-Wilk (<50 samples) and Levene tests, respectively), and therefore, a one-way analysis of variance (ANOVA) with a Tukey’s multiple range test was applied for comparative purposes using the Statistical Package for the Social Sciences (SPSS) 24.0 software package (LEAD Technologies, Inc., Chicago, IL, USA). A correlation analysis between the analytes and radical scavenging/reducing power was developed according to Pearson’s model. The significance level was set at *p* < 0.05 for all statistical analyses developed.

## 3. Results and Discussion

### 3.1. Tryptophan, Serotonin, and Melatonin Content of Grape By-Products

Both grapes and by-products are matrices with a valuable (poly)phenolic content, including phenolic acids, flavonols, flavones, anthocyanins, stilbenes, flavan-3-*ols*, and tannins [20,21]. However, although the (poly)phenols of grapes and wine have been broadly characterised in terms of their radical scavenging capacity and derived health benefits, to date, it is recognised that these are not the only bioactive molecules present in grapes and by-products. Thus, other classes of bioactive compounds characteristic of the phytochemistry of grapes have also been associated with the antioxidant activity of grapes, wine, and derived by-products. In this regard, it should be noted that winery by-products contain a valuable amino acid profile that is metabolised by yeasts and LAB as a source of nitrogen during alcoholic fermentation [22]. This utilisation of the grape amino acids by yeasts and LAB results in aroma compounds, such as high alcohols, aldehydes, esters, ketonic acids, and biogenic amines, which are to some extent responsible for the quality of grape residues as sources of bioactive phytochemicals and thereby, for their functionality [23]. Hence, during the transformation of grape amino acids by the yeasts and LAB’s metabolism, the generation of biogenic amines, such as serotonin and melatonin, significantly modulates the bioactive scope of the newly obtained co-products by diversifying the phytochemical profile [24,25] and leading to the development of their biological power through different pathways, depending on a series of molecular targets [26]. The new bioactive compounds obtained as a result of the metabolism of yeasts and LAB depend on several factors, such as the abundance of amino acid precursors in the medium, the winemaking and vinification conditions, as well as the species of microorganisms responsible for wine production (yeasts and LAB) [13]. In this frame, the study of the tryptophan content in grape by-products without contact with yeast and bacteria (grape stems) and in contact with these microorganisms during wine fermentation (grape pomace and wine lees) is of high relevance, since tryptophan is the starting point for the synthesis of bioactive compounds, such as serotonin and melatonin, and their final burden in the grape by-products derived from the winemaking process.

#### 3.1.1. Tryptophan

**Figure 2:**
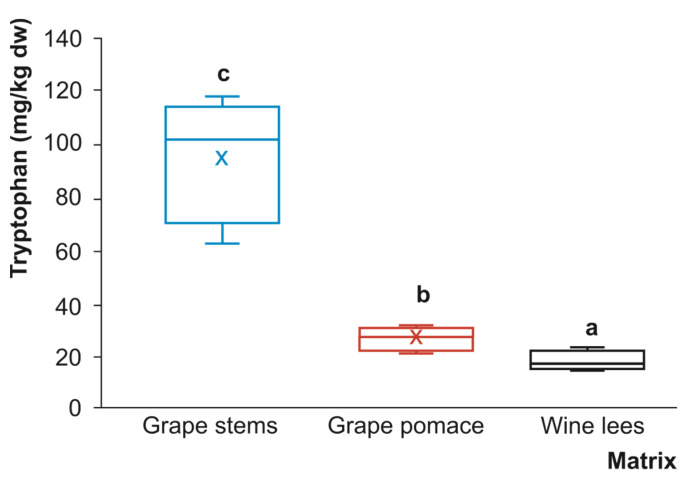
Box plots with quartiles (upper values of 75%, median values of 50%, and lower values of 25%) of tryptophan present in grape stems (blue box), grape pomace (red box), and wine lees (black box) (mg/kg dry weight (dw)) (*n* = 4 for each by-product). Boxes with a different letter within each plot were statistically different at the significant level of *p* < 0.05 according to the analysis of variance (ANOVA) and Tukey’s multiple range test.

Tryptophan is an essential amino acid with the basic function of serving as a building block for the production of physiologically necessary proteins. In addition to this structural role, tryptophan has been pointed out as a valuable antioxidant molecule [27,28]. Nonetheless, the radical scavenging functions associated with tryptophan also seem to be attributable to the hydroxylated metabolites derived from this amino acid, as tryptophan is usually metabolised before it can act as an antioxidant [29]. In this regard, to take advantage of the biological activity of tryptophan and its hydroxylated metabolites, the integration of food-/ingredient-rich tryptophan into the diet is a challenging issue, especially for those matrices with a high concentration of this essential amino acid. For this purpose, new materials need to be assessed for their content of this essential amino acid. Thus, the evaluation of winery by-products as sources of tryptophan in the present work provided mean concentrations of tryptophan that were significantly different (*p* < 0.05) in grape stems, grape pomace, and wine lees (96.28, 27.65, and 18.85 mg/kg dw, respectively) (Figure 2).

Thereby, to the best of our knowledge, this study constitutes the first description of the quantitative occurrence of tryptophan in a range of solid and semi-solid winery by-products. According to the above-mentioned numerical data, these differences are in favour of grape stems, which on average exceed by ~3.5 and ~5.0-fold times, respectively, the concentrations observed in grape pomace and wine lees. In this sense, it has been emphatically asserted that the cellular and biochemical traits of a given plant material condition the transformation of tryptophan into indoleamines via two main pathways that are governed by specific enzyme kinetics. One is the tryptophan > tryptamine > serotonin > N-acetylserotoninotonin > melatonin pathway, which is associated with normal plant growth conditions. The other pathway includes the following transformation flux: tryptophan > tryptamine > serotonin > 5-methoxytryptamine > melatonin, which takes place during tissue senescence when plants produce high concentrations of serotonin [30]. Depending on the physiological state of the plant tissue, the subcellular location of the tryptophan derivatives varies [31], thus conditioning their bioaccessibility, bioavailability, and biological functions. Moreover, the different concentrations of tryptophan in grape stems and pomace, as well as in wine lees, could indicate modifications resulting from the metabolism of yeasts and LAB present in grape pomace and wine lees, especially concerning the wine yeast strain *Saccharomyces*, which consumes tryptophan faster than the non-*Saccharomyces* ones [8]. This factor should be considered to understand the metabolism of tryptophan and the production of bioactive compounds, such as the indoleamines serotonin and melatonin, among others. According to these circumstances, the remaining tryptophan level in grape pomace and wine lees could be caused by the failure of some yeast strains to use tryptophan in the final stages of fermentation [8]. Furthermore, previous studies have suggested that the combination of yeast strain and grape variety or cultivar may play a decisive role in the production of the final tryptophan content, and ‘Monastrell’, the variety used in this study, was found to have the highest tryptophan content [32].

#### 3.1.2. Serotonin

**Figure 3:**
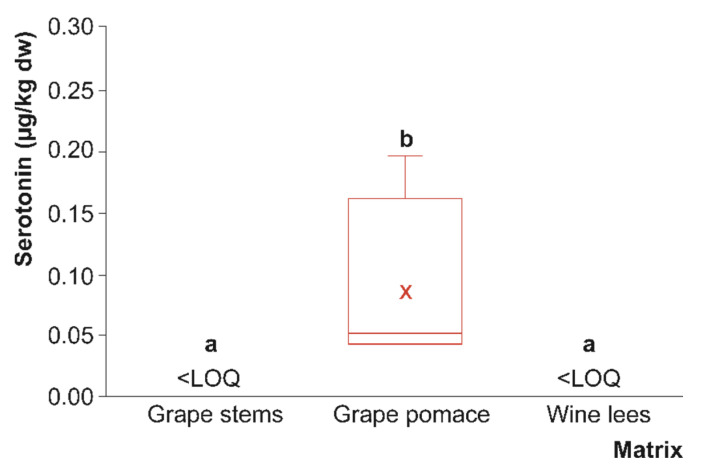
Box plots with quartiles (upper values of 75%, median values of 50%, and lower values of 25%) of serotonin present in grape stems, grape pomace (red box), and wine lees (μg/kg dry weight (dw)) (*n* = 4 for each by-product). Boxes with a different letter within each plot were statistically different at the significant level of *p* < 0.05 according to the analysis of variance (ANOVA) and Tukey’s multiple range test. LOQ, limit of quantification (500 pmol/L).

As referred to before, tryptophan is metabolised in plant material and derived co-products, as well as by humans after dietary consumption, giving rise to highly relevant bioactive indoleamines (serotonin and melatonin). As mentioned above, serotonin is a phytochemical with important radical scavenging, immunological, and neuroactive biological properties that has been detected and identified in a broad range of plant-based foods [33]. Nevertheless, as far as we know, the quantitative presence of serotonin in grape by-products or other plant by-products has not been properly described in the literature, which is an important constraint for the understanding of the functional capacity of these materials. In this respect, when grape stems, pomace, and wine lees were evaluated for their serotonin content, low concentrations (or almost non-existent) were found. Thus, only grape pomace displayed a concentration higher than the limit of quantification (0.086 µg/kg dw, on average), while it could not be quantified in grape stems or wine lees (Figure 3).

The serotonin content recorded in grape pomace was in agreement with a previous study, which reported levels of this compound ranging from 0.085 to 0.242 µg/kg in various cultivars of sweet cherries [34]. In grapes, mean concentrations of serotonin ranging from below the method’s detection limit (<LOD) up to 2.28 µg/L in the indoleamine extract have been reported [35]. Furthermore, Murch et al. described its erratic presence in part of the grapes analysed (30–35%), where concentrations up to 10.000 µg/kg were observed [24]. Despite these descriptions, relatively little is currently known about the temporal profiles of this metabolite during the fruit ripening or fermentation processes [25], which could be the origin of the variation in the serotonin levels reported. In this study, the low levels of serotonin recorded may be a consequence of the lack of proper metabolism of tryptophan to serotonin in grape stems and, secondarily, of yeast and LAB metabolism, which complete the metabolism of tryptophan to produce melatonin in grape pomace and wine lees, depleting the amount of serotonin tentatively formed as an intermediate metabolite.

#### 3.1.3. Melatonin

**Figure 4:**
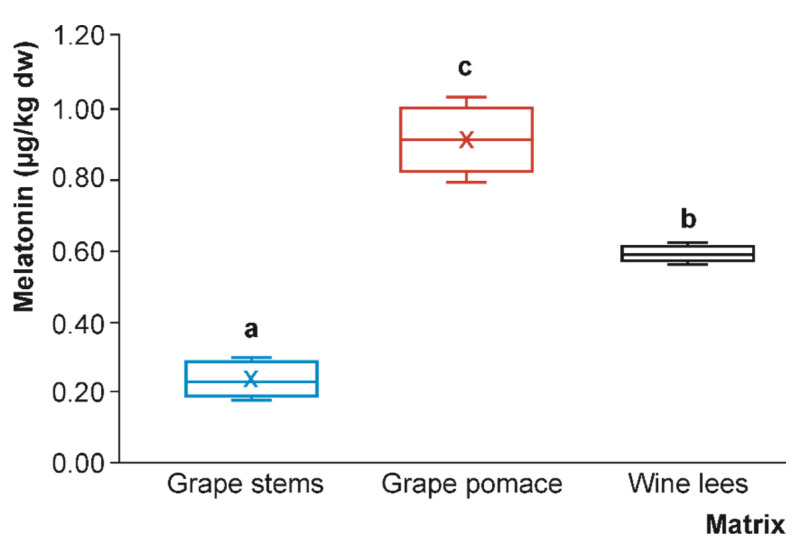
Box plots with quartiles (upper values of 75%, median values of 50%, and lower values of 25%) of melatonin present in grape stems (blue box), grape pomace (red box), and wine lees (black box) (μg/kg dry weight (dw)) (*n* = 4 for each by-product). Boxes with a different letter within each plot were statistically different at the significant level of *p* < 0.05, according to the analysis of variance (ANOVA) and Tukey’s multiple range test.

Regarding the melatonin content in plant-based foods in general, but specifically in grapes and their co-products (wine, vinegar, grappa, or juice), this metabolite was first described in grapes in 2006 as a secondary metabolite produced by the shikimate pathway [26]. However, to the present date, there is still a gap in knowledge about the occurrence of melatonin in winery by-products, which was explored for the first time in the present work. Thus, winery by-products displayed the following (significantly different at *p* < 0.05) decreasing order of concentration: grape pomace (0.902 µg/kg dw) > wine lees (0.586 µg/kg dw) > grape stems (0.234 µg/kg dw) (Figure 4).

On this matter, previous descriptions in the literature regarding melatonin levels in plant-based foods described concentrations from 10 to 20-fold higher than serotonin (considering only similar matrices such as grapefruit) [25]. In this sense, in grape pomace, the only grape by-product in which both serotonin and melatonin indoleamines were found in the present characterisation, the concentration of melatonin was almost 11 times higher than that of serotonin (0.902 vs. 0.086 µg/kg dw, respectively). As mentioned above, melatonin was not only in the berries of most cultivars of wine grape (*Vitis vinifera* L.), ranging from 0.005 to 440.0 µg/kg dw, in good agreement with the concentrations found in grape stems, grape pomace, and wine lees in the present study (0.234–0.902 µg/kg dw), but also in several grape-related foodstuffs and co-products, such as white and red wines (0.050–400.0 ng/mL), grape juice (~0.500 ng/mL), grappa (grape pomace brandy; ~0.300 ng/mL), and grape vinegar (0.110–0.130 ng/mL) [13]. However, as previously described for serotonin, the final concentration of melatonin is closely related to different physiological traits, production effects, and agroclimatic conditions that influence the biochemical characteristics of the plant material [25]. In this regard, previous research has reported that melatonin synthesis occurs during the alcoholic fermentation of several plant-based foods, such as grapes, malt, orange juice, and pomegranate [36,37,38]. According to the accepted effect of fermentation processes on melatonin formation in natural tryptophan sources, the higher melatonin content of these matrices compared to grape stems should be related to the metabolic characteristics of yeasts and LAB. Although several studies to date have ascertained that melatonin synthesis is highly dependent on the yeast strain/species and also on the metabolic state of the cell, no definitive conclusions have been reached as to the role of the different factors involved in the rise of melatonin content [39]. Therefore, further research is needed to gain a full understanding of the exact mechanism responsible for triggering or enhancing melatonin synthesis, including the potential formation of melatonin metabolites such as 2-hydroxymelatonin during fermentation [8]. Even so, the description of serotonin and melatonin in these oenological by-products is of great relevance, not only for their recovery and sustainable valorisation but also for obtaining new food ingredients with improved quality and functionality.

### 3.2. Antioxidant Activity

Several methods have been developed to assess the antioxidant capacity of natural compounds and plant or food extracts rapidly and successfully. The different methods basically work by two different mechanisms: transferring either an electron (ET) or a hydrogen atom (HAT) [40]. All these methods are based on chemical reactions and the evaluation of the kinetics or the final equilibrium state. Accordingly, in the present work, the in vitro antioxidant capacity of the samples extracted for tryptophan, serotonin, and melatonin analyses was determined by three complementary methods: the ORAC assay, which involves HAT from the antioxidant to peroxyl radicals, reflecting physiological relevance [41]; the FRAP assay for reduction of the Fe(III)/tripyridyltriazine complex, based on an electron transfer (ET) from the antioxidant; and the ABTS^•+^ assay, which includes both HAT and ET mechanisms [42]. The utility of these methods has been demonstrated, as several studies have shown a positive association between high consumption of plant products with high antioxidant capacity (dietary ORAC units per day) and lower levels of inflammatory cytokines and oxidative stress biomarkers, as well as a lower risk of chronic diseases [40,43,44]. Thus, in the present work, ORAC, ABTS^•+^, and FRAP assays were selected as complementary methods to measure antioxidant activity due to their different bases for chemical reactions [42].

Oxidative stress caused by ROS plays an active role in the pathophysiology of non-communicable diseases, such as diabetes, obesity, and atherosclerosis. Dietary antioxidants are therefore essential to reduce the risk of these degenerative conditions [44]. Given the relevance of oxidative stress in various pathophysiological issues, as well as the need to identify natural compounds with an adaptive advantage, the use of different in vitro methods to evaluate the potential of dietary antioxidant phytochemicals, as well as their bioaccessible and bioavailable derivatives, against ROS is mandatory [42,44].

To the best of our knowledge, very few works have reported and/or compared the antioxidant capacities of tryptophan, serotonin, and melatonin from plant samples. For example, the antioxidant capacity of tryptophan (~700 µM) from the lotus plant (*Nelumbo nucifera* Gaertn) was demonstrated using the inhibition tests of lysis of erythrocytes and lipid peroxidation [27]. In addition, very recent work has shown a weak correlation between the content of these indoleamines (tryptophan, serotonin, and melatonin) and the total antioxidant capacity of aqueous extracts of corn and pea samples [45]. Apart from plant foods, Nayak and Buttar also showed tryptophan as an antioxidant compound in human milk (7986 µmol TE/g by ORAC assay) [28]. Therefore, this work contributes information to this little-studied field of the antioxidant capacity of these indoleamines extracted from plant sources. Our results showed that grape stems were the samples with the highest antioxidant capacity, regardless of the extraction method (for tryptophan or serotonin/melatonin analyses) or the antioxidant assay used (Table 2).

The different winery by-products were compared in terms of the results obtained for each antioxidant (FRAP, ABTS^•+^, or ORAC) and extraction method (tryptophan or serotonin/melatonin), since higher results were found in those extracted for tryptophan, ranging from 18 to 363 mmol TE/kg dw, than for serotonin and melatonin evaluations, ranging from 15 to 154 µmol TE/kg dw.

The antioxidant capacity of grape stem samples extracted with the method developed for tryptophan was about 8-fold higher and 3-fold higher compared to wine lees and grape pomace, respectively, for all antioxidant methods used. This fact could be attributed to the higher tryptophan content in grape stems compared to the other by-products (Figure 2). Tryptophan, as well as its derived metabolites, has been reported to play an important role as a ROS scavenger and activator of antioxidant systems in the body, making it an interesting functional food component [29]. However, the presence of other hydrophilic compounds in the samples seems to influence the antioxidant capacity, as wine lees showed higher results compared to grape pomace, although its tryptophan content was significantly lower (Table 2). Recent results from our group described a higher total content of (poly)phenolic compounds in grape stems compared to grape pomace and wine lees [46]. Thus, although the extraction method for tryptophan is not the optimal choice for the extraction of phenolic compounds, a part of them could be released during the specific process applied for the analytes focused on the objectives of the present work, contributing to the overall antioxidant capacity recorded (Table 2).

When these by-products were extracted for serotonin and melatonin analyses, no significant differences in antioxidant capacities were found between grape pomace and wine lees, regardless of the antioxidant method used (Table 2), although grape pomace showed higher levels of serotonin and melatonin than wine lees (Figure 3 and Figure 4). Thus, the presence of serotonin and melatonin in the samples (ranging from 0.02 to 0.19 and from 0.2 to 1.0 µg/kg dw, respectively) does not seem to be responsible for the main antioxidant capacity of these by-products. This fact could be attributed to the presence of other hydrosoluble bioactive compounds extracted by this method, such as (poly)phenols. However, serotonin and melatonin have been widely described as highly potent endogenous radical scavengers by H-atom transfer and electron donors in humans, as well as stimulators of antioxidant enzymes [33,47].

The correlations between the three methods were positively high in the r^2^ range of 0.969–0.995 (*p* < 0.01), as suggested by other authors for the analysis of samples with high content of hydrophilic compounds, such as (poly)phenols from fruits (blueberries, peaches, and plums). However, these methods are more reproducible for hydrophilic extracts, as Thaipong et al. reported that ABTS^•+^ and FRAP were negatively correlated when analysing the lipophilic antioxidant capacity of β-carotene [42]. According to other authors, the ORAC method seemed to be more sensitive to the antioxidant activity of the samples, providing the highest results [48]. Thus, this method could be considered the most effective for quantifying the antioxidant capacity in plant samples with low content of antioxidant compounds, also because of its biological relevance to the in vivo antioxidant efficacy. The importance of using different antioxidant capacity methods lies in the different chemical reactions involved as well as the different sensitivities established in each method, which could lead to an underestimation of the antioxidant capacity of the sample, so it is advisable to use at least two antioxidant methods to obtain reliable results [49]. Therefore, the three selected methods provided us with information on different aspects of the antioxidant capacity of these samples extracted for the quantification of tryptophan, serotonin, and melatonin, providing a complete picture of their antioxidant behaviour, although their respective relevance in biological systems should still be further clarified.

In order to determine the relative contribution of tryptophan, serotonin, and melatonin to the total radical scavenging and reducing capacity of winery by-products, their antioxidant capacity was analysed as pure compounds at different concentrations to improve the interpretation of their potential without the interference of additional compounds present in the whole tryptophan and serotonin/melatonin extracts being evaluated. Different concentrations of these compounds were evaluated based on the different antioxidant capacity methods used. Interestingly, only serotonin (5, 10, 25, 50, and 100 µM) showed antioxidant capacity in the FRAP and ABTS^•+^ assays, with a dose-dependent response observed as shown by the linear equation presented in Table 3 for the concentrations studied. Serotonin showed a higher antioxidant capacity than the standard Trolox, with 100 µM serotonin providing 162.9 and 182.6 µM TE in the FRAP and ABTS^•+^ assays, respectively. On the other hand, in terms of ORAC-based antioxidant activity, the three pure compounds (tryptophan, serotonin, and melatonin) exhibited significant antioxidant capacity at the concentrations of 1.25, 2.50, 5.00, and 10.00 µM (Table 3). The responses of the three compounds followed a linear equation in the range of 1.25 to 10 µM, as shown in Table 3. However, higher concentrations of the three compounds (25 µM) were above the upper limit of quantification of the present ORAC method. Nevertheless, the results showed that tryptophan, serotonin, and melatonin may contribute to the antioxidant capacity of the samples, giving them higher bioactivities compared to the reference standard, Trolox.

According to our results from the ABTS^•+^ and FRAP assays, serotonin demonstrated a higher antioxidant capacity in vitro (by radical scavenging and cupric ion reducing ability) compared to melatonin due to its phenolic group [4]. Other authors showed antioxidant capacity by melatonin in the ABTS^•+^ method at lower concentrations compared to our work (4–20 µM) [50], and thereby, further studies are needed to explore differences in the antioxidant capacity methods and responses. Nevertheless, when analysing the correlation between these compounds and the antioxidant capacity assessed by ABTS^•+^, FRAP, and ORAC methods, the only compound that showed a significant positive correlation was tryptophan (ABTS^•+^, r^2^ = 0.891 at *p* < 0.001 (***); FRAP, r^2^ = 0.885 at *p* < 0.01 (**); and ORAC, r^2^ = 0.854 at *p* < 0.01 (**)). However, serotonin and melatonin showed a weak correlation with the antioxidant capacity, which could be due to the low levels of these indoleamines in the samples. In this context, recent studies also showed a low correlation between serotonin and melatonin, as well as between both of them and the total antioxidant capacity of corn and pea samples. However, contrary to what we found, the authors did not find a positive correlation between tryptophan content and total antioxidant capacity [45]. Further studies should therefore be carried out to highlight the antioxidant capacity provided by these compounds in plant-based foods, depending on the concentrations in which they are found.

## 4. Conclusions

The evaluation of tryptophan, serotonin, and melatonin levels in grape stems, grape pomace, and wine lees developed in the present work reveals concentrations in the range of other plant-based foods or beverages as discussed above. This completes the phytochemical profile already described for these materials, which have been mainly focused on their (poly)phenolic burden and, as a result, the potential biological effects in the frame of a rising number of pathophysiological conditions beyond the prevention of oxidative stress, according to their capacity to regulate a range of neuroendocrine, cardio-respiratory, thermoregulatory, and immune functions, closely related to the regulation of the sleep-wake cycle or pain sensitivity. It is also important to note that the three residues assessed displayed complementary profiles of tryptophan and its derivatives, with the precursor being more abundant in grape stems and the derivatives in grape pomace and wine lees, which would allow a combined formulation of the three residues to obtain the best results in terms of bioactivity and health promotion. Concerning the radical scavenging capacity demonstrated by these compounds, which is the focus of the functional potential characterised in the present work, they would develop joint functions with (poly)phenols, preventing the deleterious effect of ROS on several components of living cells (DNA, membrane lipids, and/or cytosolic proteins). Interestingly, the assessment of authentic standards in the range of concentrations found in the tryptophan or serotonin/melatonin extracts provides solid evidence concerning the actual interest of these compounds as functional molecules related to human health and well-being. Furthermore, it is important to emphasise that these functions may be developed by specific molecular mechanisms that are different from those triggered by (poly)phenols, which would inform on potential synergies between both classes of bioactive compounds, reinforcing the valorisation potential of winery by-products. As an overall consequence of this complementarity, the phytochemical and functional characterisation developed in the present work leads to envisage new valorisation alternatives for the winery by-products as new food ingredients with the potential to be used to improve the nutritional quality and functionality of newly developed added-value co-products in the food and cosmetic industries.

## Figures and Tables

**Table 1:** UHPLC-ESI-QqQ-MS/MS parameters for identifying and quantifying tryptophan, serotonin, and melatonin in grape stems, pomace, and wine lees.

Compound	MRM Quantitative Transition	Fragmentor (V)	Collision Energy (eV)	ESI Mode	LOD (pmol/L)	LOQ (pmol/L)
Tryptophan	375.0 > 171.2	120	0	Positive	31	31
Serotonin	177.0 > 159.9	90	0	Positive	250	500
Melatonin	233.0 > 174.3	90	0	Positive	31	61

ESI, electrospray ionization; MRM, multiple reaction monitoring.

**Table 2:** Antioxidant capacity in grape stems, grape pomace, and wine lees samples extracted for tryptophan and serotonin/melatonin analysis.

Method	By-Product	Extraction for Tryptophan Analysis (mmol TE/kg dw)	Extraction for Serotonin and Melatonin Analysis (µmol TE/kg dw)
FRAP			
	Grape stems	142.86 ± 8.04 a	49.04 ± 2.69 a
	Grape pomace	19.05 ± 0.80 c	18.52 ± 0.85 b
	Wine lees	53.17 ± 1.00 b	18.80 ± 0.36 b
ABTS^•+^			
	Grape stems	166.72 ± 6.77 a	59.53 ± 2.31 a
	Grape pomace	17.84 ± 0.99 c	19.31 ± 1.73 b
	Wine lees	45.96 ± 1.02 b	15.46 ± 1.22 b
ORAC			
	Grape stems	363.24 ± 11.0 a	153.77 ± 13.03 a
	Grape pomace	36.41 ± 1.58 c	46.22 ± 2.56 b
	Wine lees	128.33 ± 13.0 b	56.52 ± 4.27 b

Results are expressed as mean ± SD (*n* = 4). TE: Trolox equivalents. The statistical analysis was performed independently within the samples extracted for tryptophan, serotonin, or melatonin. Values followed by different letters (a–c) within each extraction method and antioxidant activity method are significantly different according to a one-way analysis of variance (ANOVA) and multiple range test of Tukey at *p* < 0.05.

**Table 3:** Antioxidant capacity of the pure compounds tryptophan, serotonin, and melatonin expressed as µM Trolox equivalent (TE).

Method	Standard Concentration (µM)	µM TE	Linear Equation	R^2^
Serotonin				
FRAP	100	162.9 ± 1.4	y = 1.62x + 2.4	0.999
ABTS^•+^	100	182.6 ± 3.8	y = 1.74x + 14.5	0.988
ORAC	10	42.5 ± 0.3	y = 4.75x − 3.9	0.999
Melatonin				
FRAP	100	N.d.	N.d.	N.d.
ABTS^•+^	100	N.d.	N.d.	N.d.
ORAC	10	45.8 ± 0.9	y = 4.20x − 8.4	0.998
Tryptophan				
FRAP	100	N.d.	N.d.	N.d.
ABTS^•+^	100	N.d.	N.d.	N.d.
ORAC	10	33.5 ± 2.7	y = 5.02x − 4.0	1.000

Samples of pure compounds were analysed in quadruplicate (*n* = 4; mean ± SD). N.d., not detected. The linear equations represent the linearity of the response of the antioxidant capacity for the tested concentrations of the three compounds: 5, 10, 25, 50, and 100 µM for the FRAP and ABTS methods, and 1.25, 2.50, 5.00, and 10.00 µM for the ORAC assay.

## Data Availability

Data is contained within the article.
